# Field evaluation of the efficacy of ovillanta ovitrap towards *Aedes* spp. control in a dengue endemic area of Kerala, India

**DOI:** 10.1016/j.parepi.2026.e00516

**Published:** 2026-05-28

**Authors:** Abidha Suresh, Shriram Ananganallur Nagarajan, Jessu Mathew, Amju Kolakattum Poyilthazham, Sonia Thankachy, Prasanta Saini, Kannan Thiruvengadam, Pradeep Kumar Narendran, Ashwani Kumar

**Affiliations:** a ICMR-National Institute for Vector Control Research, Field Station, Kottayam 686003, India; b ICMR-National Institute for Vector Control Research, Puducherry 605006, India

**Keywords:** Ovillanta, *Aedes aegypti*, Dengue, Immatures, Vector

## Abstract

A small-scale field trial was conducted to understand the efficacy of the ovillanta ovitrap against *Aedes* vector population in a dengue endemic area of Kerala state. A total of 40 ovillantas were installed in experimental area, and the immature density of *Aedes* vectors were monitored over 14 months. Pre- and post- intervention entomological evaluations were conducted in both the experimental and control areas to assess the density of *Aedes* spp. An increasing trend in the weekly average immature density was observed, indicating the efficacy of the trap to lure gravid vectors to oviposit, which reached a peak at 39th week. Forty ovillantas enabled to destroy more than 100,000 immatures, during the 53-week study period, thus preventing the emergence of second generation of vectors. There was a significant reduction in the adult and immature indices of the experimental area, compared to the control area. The results of the study suggest that the ovillanta is a simple and portable tool that may be suitable for community use in *Aedes* vector control.

## Graphical abstract

**Figure:**
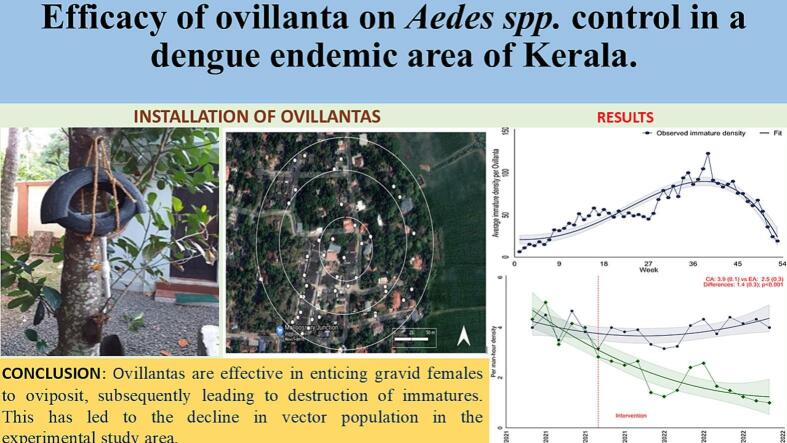

## 1 Introduction

Dengue has gained prominence as an essential arboviral disease affecting more than 100 countries in the tropical and subtropical regions of the world (Bhatt et al., 2013). Recent studies estimate that 3.6 billion people are at risk of dengue infection, with over 230 million infections globally (WHO, 2020a). Dengue fever is a tremendous public health concern in India, with the first major outbreak in Delhi in 1996, reporting 8900 cases and 4.2% deaths (Dar et al., 1999). During 2021, a total of 1,93,245 dengue cases and 346 deaths were reported in India. In 2022, a total of 233,251 cases and 303 deaths due to dengue fever were reported in the country, suggesting a 20% increase in dengue cases and a 12.7% decrease in mortality (NCVBDC, 2024). Kerala was severely affected by dengue during the major dengue outbreak in 2017, contributing to more than 13% of the total cases in the country (Kumar et al., 2019). In 2022, the dengue incidence in Kerala was 4432 with 29 deaths, whereas during the 2017 outbreak period, 21,993 cases and 165 deaths were reported (DHS Kerala, 2022). There was 79.8% reduction in dengue cases and 82.4% reduction in fatalities, indicating an overall decreasing trend in dengue incidences and deaths. For the past few decades, *Aedes aegypti* has been a significant public health concern in both tropical and subtropical countries. This is attributed to its capacity to transmit various globally significant arboviral diseases, primarily dengue, chikungunya and zika.

**Fig. 1:**
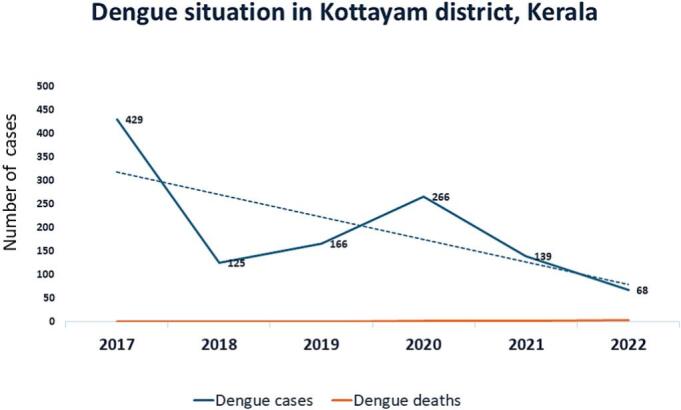
Dengue cases and deaths in Kottayam District, Kerala from 2017 to 2022.

Kottayam district of Kerala was reported as the epicentre of dengue with all four dengue virus (DENV) circulating serotypes and *Ae. aegypti* is the predominant vector (Kumar et al., 2013; Abidha et al., 2021). Cyclic dengue epidemics have been occurring in Kerala since 2001, although the first dengue report was recorded in 1997, from Kanjirappally taluk. In 2017, Kottayam district harboured 429 cases, with primary urban foci in Kanjirappally (Kanjirappally taluk) and Malloossery (Kottayam municipality). During 2022, 139 cases and two deaths were reported from Kottayam district (DHS Kerala, 2022). The district also showed a reduction trend in dengue incidences during 2017–2022 (Fig. 1). Entomological surveys conducted by the Indian Council of Medical Research -National Institute for Vector Control Research (ICMR-NIVCR) in these areas from 2014 to 2019, indicated *Ae. aegypti* as the predominant vector (95.2% and 99.5% vector prevalence, in Kanjirappally and Malloossery, respectively). Other mosquito species collected included *Culex quinquefasciatus* and *Armegeres subalbatus*. The key breeding habitats of this species are water storage containers (85.79%), which are maintained in each household in the urban areas (Vector Control Research Centre Puducherry. Annual Report, 2015). This could be attributed to the acute water deficit during the summer season and the community's resort to water storage practices, which leads to vector proliferation. The breeding sites of *Aedes* mosquitoes, apart from water storage containers, included unused buckets, plastic containers, cement tanks, discarded vessels, the rims of flower pots, flower vases, and glass bottles containing ornamental plants (Arunachalam et al., 2010). Earlier studies conducted in the same area also reported that the presence of immature of *Ae*. *aegypti*, *Ae*. *albopictus*, *Cx*. *quinquefasciatus* and *Ar*. *subalbatus* occurred in coconut shells, discarded tyres, tree holes, and various discarded artificial containers (Balasubramanian and Nikhil, 2013). (See Table 1.)

**Table 1:** Entomological survey results of study areas (Pre and post ovillanta installation).

Months	Arm 1 (Nirmithi colony, Malloossery)					Arm 2 (Nachi colony, Kanjirappally)				
	PMHD	HI	PI	CI	BI	PMHD	HI	PI	CI	BI
Mar 21⁎, #	4.33	72.92	91.66	41.18	116.66	4.00	68.75	95.83	38.39	114.58
Apr 21⁎, #	5.00	75.0	87.75	43.51	118.75	4.50	70.83	89.58	40.8	106.25
May 21⁎, #	3.33	56.25	47.91	29.86	89.58	3.50	52.08	41.66	26.67	85.41
Jun 21	4.16	70.83	77.08	43.65	114.58	4.66	72.91	72.91	42.06	110.41
Jul 21	4.00	64.58	50.00	40.11	104.16	3.83	66.66	52.08	38.23	108.33
Aug 21	2.83	58.33	47.91	29.62	66.66	3.16	62.5	54.16	35.12	81.25
Sep 21	2.66	43.75	22.91	26.25	43.75	4.00	58.33	47.91	38.88	87.5
Oct 21	2.50	20.83	20.83	21.73	20.83	3.83	41.66	35.41	32.63	64.58
Nov 21	2.66	35.41	18.75	24.05	39.58	3.91	47.91	41.66	35.13	81.25
Dec 21	1.41	29.16	16.66	19.2	33.33	3.33	43.75	39.58	27.14	79.16
Jan 22	1.25	18.75	12.5	10.84	18.75	3.16	37.5	41.66	25.0	66.66
Feb 22	1.50	22.92	14.58	16.87	29.16	3.25	39.58	56.25	31.53	75.0
Mar 22#	2.41	27.08	20.83	18.82	33.33	4.08	64.58	87.5	36.49	104.17
Apr 22#	2.58	35.41	33.33	20.87	39.58	4.33	68.75	93.75	38.03	112.5
May 22#	1.66	20.83	14.58	14.81	25.0	3.75	54.17	58.33	30.71	89.58
Jun 22	1.49	18.75	16.66	19.23	31.25	4.41	68.75	66.66	38.46	104.16
Jul 22	1.24	16.66	12.5	14.28	20.83	4.16	62.5	47.92	37.84	87.50

PMHD: Per man hour density, HI: house index, PI: pupal index, CI: container index, BI: Breteau index.

⁎ Entomological collections prior to Ovillanta installation.

# Summer months in the study area.

Currently, vector control is the most practically feasible approach for preventing and controlling vector borne arboviral diseases (Dambach et al., 2024). As there is no effective vaccine to prevent these diseases, mosquito management including chemical, biological and physical strategies, is the primary practice used to control them (Tawatsin et al., 2021). Current vector control strategies for dengue primarily emphasize source reduction techniques.

Several countries are strengthening their research efforts to design and develop effective and sustainable intervention strategies for controlling *Aedes* populations (Bouyer, 2024; Cilek et al., 2024; Mulderij-Jansen et al., 2022). The widely preferred surveillance mechanisms include trapping devices, such as BG-Sentinel traps and ovitraps, which primarily target eggs (Regis et al., 2008) or gravid females (Barrera et al., 2014). In contrast, modified lethal ovitraps and autocidal gravid ovitraps (AGO) traps are specifically engineered to reduce mosquito populations by targeting gravid females and interrupting the reproductive cycle and are being used for vector surveillance (Aguilar-Durán et al., 2024). When deployed at adequate coverage and density, these lethal trap systems have demonstrated measurable reductions in *Ae. aegypti* abundance and may contribute to integrated vector management strategies. These devices claim to have greater potential and efficacy in reducing vector populations which could ultimately result in an interruption of DENV transmission (Mackay et al., 2013; Regis et al., 2013). Dengue vector control strategies have expanded in recent years, with mass trapping increasingly recognized as an emerging approach (Barrera, 2022). Gravid ovitraps target gravid females which attempt to oviposit inside the trap, so that mass ovitrapping tends to reduce the fecundity of the *Aedes* population. Continuous application in the long run enables the reduction of vector density, which may also include the infective ones (Ulibarri et al., 2017).

Recently, novel strategies for *Aedes* vector control have been developed, including modified AGO traps which include the CDC-AGO (Centres for Disease Control and Prevention-Autocidal Gravid Ovitrap) and ovillanta. Both these traps have already been evaluated during recent Zika outbreaks in South America and were found to be effective in controlling the *Aedes* population. Among these, ovillanta (“*ovi*” means egg in Latin and “*llanta*” means tyre in Spanish), fabricated from used automobile tyres, is simple, economical, portable, easy to maintain, environmentally safe, and community friendly (Ulibarri et al., 2017).

Dr. Gerardo Ulibarri of Laurentian University, Ontario, is the inventor of the ovillanta ovitrap. He first evaluated its efficacy in Guatemala by comparing 84 ovillantas with standard ovitraps, which use black cups to attract gravid females for oviposition. The study found ovillantas to be seven times more effective than standard AGOs (Ulibarri et al., 2017). Discarded tyres contribute to about 30% of breeding habitats of dengue vectors in urban areas (Abílio et al., 2018). The water from the ovillanta blended with hay infusion serves as a natural attractant. According to earlier reports, ovitraps baited with 10% hay infusion yielded 8 times more *Ae. aegypti* eggs than CDC ovitraps containing tap water (Polson et al., 2002). Repeated recycling over a period of time, enhances its luring capacity, due to the accumulation and concentration of natural pheromones, released by gravid vectors, in the water. This optimisation of inherent natural cues entices more gravid mosquitoes to alight on it. Thus, ovillantas made from used tyres serve as a suitable attractant to gravid vectors (Ulibarri et al., 2017).

This strategy helps to eliminate the immature stages, prevent the emergence of the second generation of vectors, and subsequently interrupt dengue transmission in the area, by reducing the vector density. After evaluation of its efficacy, it could be adopted as a sustainable vector control tool by the community itself. Hence, a small-scale field trial study was undertaken to assess the effectiveness of this simple, economically and environmentally friendly strategy utilizing ovillantas, in an endemic area of Kottayam municipality, for its utility, in the controlling the *Aedes* vector population.

## 2 Materials and methods

### 2.1 Study sites

**Fig. 2:**
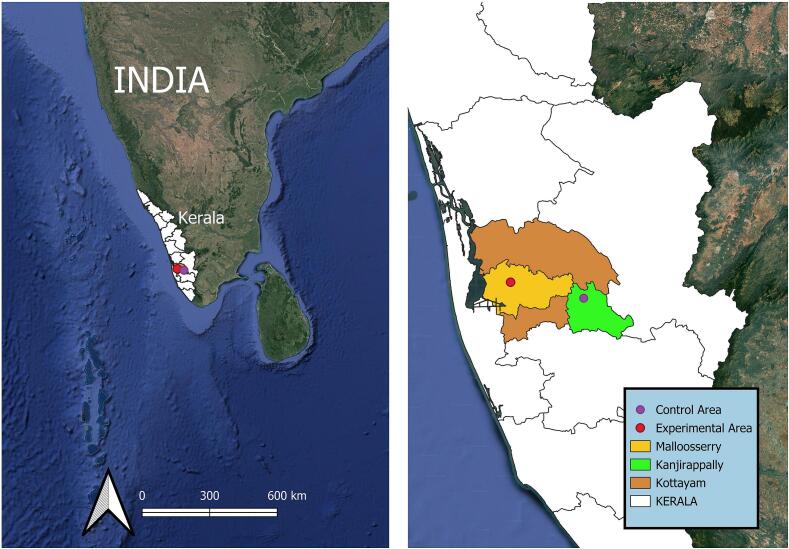
Map of Kerala state in India showing the experimental area and control area.

This study utilized a non-randomized, two-arm quasi-experimental design to facilitate a robust comparison between two geographically distinct areas while ensuring they are matched on critical ecological and demographic variables. The experimental area (EA) selected for the trial was Nirmithi colony, a residential urban settlement in ward 10 (Malloossery), belonging to Kottayam municipality, with a population of 1215 persons and 290 households. This area contributes to dengue cases each year, with an incidence rate of 6.32 in 2017. For the two-arm comparison, a non-randomized purposive allocation method was used to select the control area (CA) based on comparable baseline characteristics. The CA selected for the study was Nachi colony, ward 9, located in Kanjirappally taluk, with an urban population of 1, 352 persons and 320 houses (Fig. 2). The dengue incidence rate was 5.18 during 2017 (Source: dhs.kerala.gov.in).

The topography, house clustering, water scarcity and storage practices were found to be similar in both areas. During the acute summer season, residents commonly resort to water storage to cope with the shortage, which facilitates the proliferation of *Aedes* spp., particularly *Ae. aegypti*. Non-biodegradable containers used for water storage serve as significant breeding sites for larvae during the dry season (Balasubramanian et al., 2015).

### 2.2 Intervention with Ovillanta

**Fig. 3:**
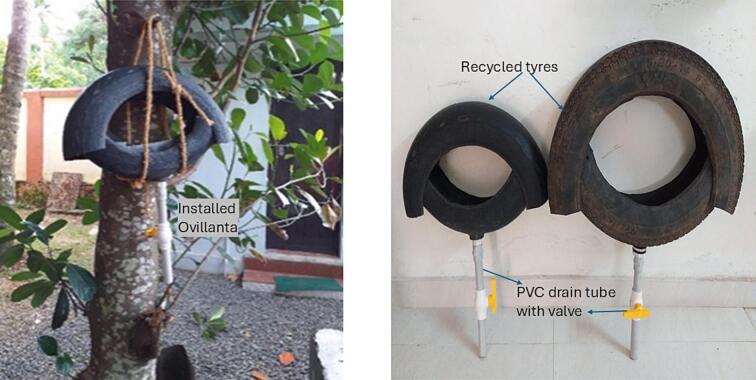
a. Installed ovillanta in the study area; b. Ovillanta made from recycled tyres.

Ovillanta was constructed from two 20-in. (50 cm) sections of discarded automobile tyres, fitted with a PVC wash tube and a valve to drain the water (Fig. 3). Hay infusion was prepared by soaking 125 g of dried hay straw in 15 l of clean water in a closed plastic bucket for seven days. A 10% hay infusion, mixed with two litres of clean, well water, served as a natural attractant (Polson et al., 2002). The ovitrap was equipped with two filter paper strips, measuring 15 cm × 10 cm, on either side of the apparatus, which served as landing niches for the gravid females to oviposit.

**Fig. 4:**
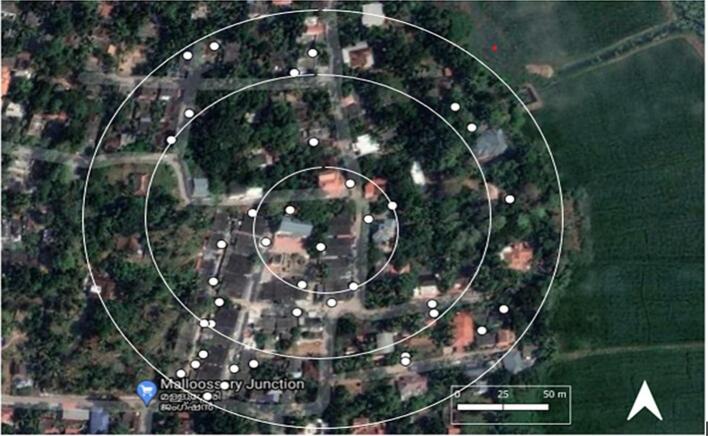
Distribution of 40 ovillantas in concentric circles in experimental area.

The study was conducted from March 2021 to July 2022. Prior to installation of traps in EA, residents were informed about the potential risks and benefits associated with using the traps. Installations were conducted voluntarily with full cooperation of the participants. One ovillanta was set up in every 7th house radially in concentric circles, from a central point and since there are 290 households, a total of 40 traps were required, taking into account the flight range of vectors and distance between the households (Fig. 4), following the sampling strategy adopted by Barrera et al. (2018). The immature density was monitored twice a week, and the water from the lower half was drained, filtered and reused in the tyre, as it may retain olfactory cues that attract gravid females for oviposition. The filtrate containing the immatures was discarded onto dry ground, preventing the emergence of the new generation of *Aedes* vectors.

### 2.3 Mosquito collections

Fortnightly entomological surveys were conducted, in accordance with the WHO standard guidelines, in both in EA and CA, prior to and after installation of Ovillantas to assess the impact of the control strategy by monitoring the adult indices (WHO, 2020b). Adult mosquito collections were conducted during the morning hours with the assistance of a torch and mechanical aspirators to estimate the per man hour vector density (PMHD). A total of 24 houses were selected in each study arm, and fortnightly adult mosquito collections were conducted in all selected households. Approximately 15 min were spent in each household, resulting in a total of 360 min (6 man-hours) of collection effort per study arm.

### 2.4 Vector indices

Wet containers were inspected for the presence of *Aedes* larvae and pupae both in domestic and *peri*-domestic areas, to determine the following larval and pupal indices (Focks, 2003):

$$\text{House index}\;\left[\mathrm{HI}\right]:\frac{\text{Infested houses}}{\text{Houses inspected}}\times 100$$

$$\text{Container index}\;\left[\mathrm{CI}\right]:\frac{\text{Containers positive}}{\text{Containers inspected}}\times 100$$

$$\text{Breteau index}\;\left[\mathrm{BI}\right]):\frac{\text{Containers positive}}{\text{Houses inspected}}\times 100$$

$$\text{Pupal index}\;\left[\mathrm{PI}\right]:\frac{\text{Number of pupae}}{\text{Houses inspected}}\times 100$$

### 2.5 Data analysis

Data were thoroughly reviewed for completeness and consistency prior to statistical analysis using STATA version 18.5 (StataCorp, College Station, TX, USA). An independent Student's *t*-test assuming equal variances was used to compare larval and adult density indices between experimental and control areas across different months. Additional comparisons were conducted for the entire study period, as well as specifically for the post-intervention phase, to assess the consistency and robustness of the intervention effects. A *p*-value<0.05 was considered statistically significant. To evaluate post-intervention temporal trends while accounting for seasonality, we employed a linear regression model incorporating Fourier terms (sine and cosine functions) based on weekly observations. The model accounted for cyclical seasonal patterns, with week number included as a continuous predictor and the Fourier terms used to adjust for annual variation in average immature mosquito density.

### 2.6 Ethics statement

This study did not involve human participants or vertebrate animals. Oral consent was obtained from household owners before conducting entomological surveys and installing ovitraps. No personal data or human specimens were collected during this study.

## 3 Results

**Fig. 5:**
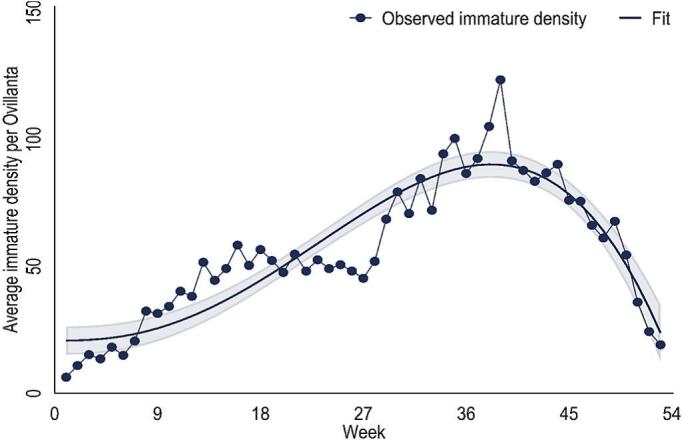
Average immature density per ovillanta in experimental area over 53 weeks.

During the pre-installation period, the entomological parameters were high in both EA and CA. Following the post-installation in EA, an increasing trend in the immature mosquito density was observed up to April 2022, after which it began to decline. The maximum average weekly immature density observed per ovillanta was 123.5, recorded during the 39th week (April 2022), indicating the effectiveness of the device in attracting gravid vectors to oviposit, as shown in Fig. 5. By the end of 53 weeks, the density had decreased to 19.12, with a corresponding reduction in both adult and larval indices in EA. A linear regression model explained 70.2% of the variation in immature density observed per ovillanta (R^2^ = 0.702), and was statistically significant (F(3,49) = 38.50, *p* < 0.001). Although a positive trend was observed for the week variable (coefficient = 0.345, *p* = 0.108), it was not statistically significant, suggesting that seasonal patterns may have overshadowed the time-based effect. Both sine and cosine terms were highly significant (*p* < 0.001), indicating strong seasonal fluctuations in mosquito density, with reductions corresponding to specific phases of the year. These results suggest that, while there was a slight increasing trend over time, seasonality was the primary driver of variation in immature density observed per ovillanta, with no significant linear trend attributable to time alone.

**Fig. 6:**
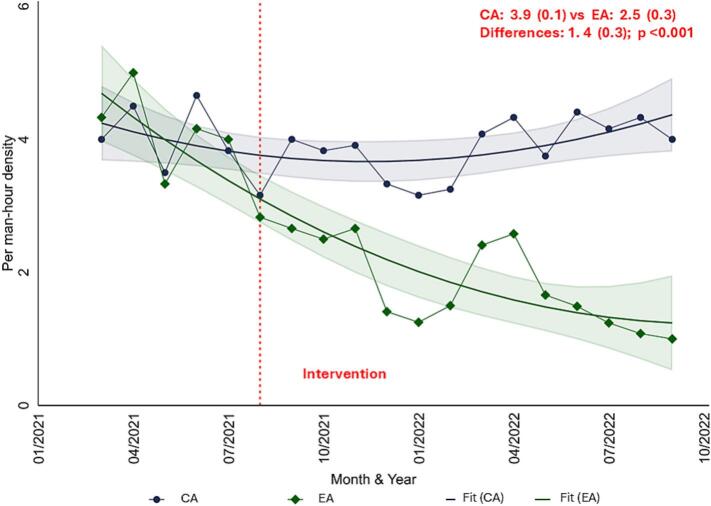
Per man hour density of adult mosquitoes in experimental area and control area pre and post ovillanta installation over the months.

**Fig. 7:**
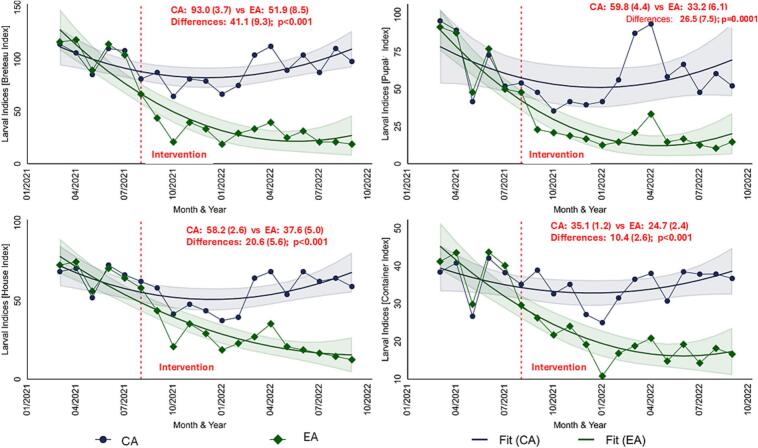
Larval and pupal indices of immatures in experimental area and control area pre and post ovillanta installation over the months.

The per man hour density (PMHD), which measures the indoor resting adult vector population, revealed a significant reduction in EA compared to CA (p < 0.001) (Fig. 6). Immature indices used to estimate the larval population also showed a significant reduction in EA, compared to CA. The PI in EA decreased from 91.66, during March 2021to 20.83 during the peak breeding season in March 2022. By the end of the experiment in July 2022, the PI in EA had further decreased to 12.5. Statistical analysis confirmed a significant reduction in PI in EA compared to CA (*P* = 0.001) (Fig. 7). Similarly, the HI, CI, BI, all exhibited significant decreasing trend in EA compared to CA (p < 0.001 for all) (Fig. 7). Overall, the strategy led to the destruction of more than 100,000 (*n* = 1,19,467) immatures during the 53 weeks study period. On average, 9956 immatures were trapped and destroyed per month. The highest immature count (4,940) occurred during peak season in the 39th week (April 2022), while the lowest count (765) was recorded in the 53rd week (July 2022), at the conclusion of the experiment.

## 4 Discussion and conclusion

Overall, field evaluation indicates that the Ovillanta ovitrap, constructed from recycled tyres, achieved higher *Aedes* egg capture and was associated with reduced adult mosquito abundance. In the current study, a significant reduction of 84.51% of immature *Aedes* mosquitoes was observed following the installation of ovillantas. A 10-month study conducted in Sayaxche, Guatemala, in 2015 demonstrated the effectiveness of ovillantas in reducing DENV transmission. Researchers successfully destroyed approximately 18,000 *Aedes* eggs per month, nearly 200,000 in total, by deploying 84 ovillantas in households. The traps were found to be seven times more effective than standard AGOs (Ulibarri et al., 2017). The trap used in this study was modified by incorporating a 10% hay infusion with 2 l of clean water, which served as a natural oviposition attractant for gravid females. In contrast, Ulibarri's ovillanta trap involved the application of 8 ml of AE lure solution to 2 l of clean water.

To the best of our knowledge, this is the first study to evaluate the efficacy of ovillanta ovitraps in an Indian setting, specifically in a dengue endemic area of Kerala. The findings were encouraging, showing that the device was able to attract gravid *Aedes* females, facilitating oviposition, and enabling the collection and removal of immature stages. A decline in the adult vector population was also observed within the experimental areas during the study period. However, these observations primarily reflect trap performance under field conditions and this trend cannot be directly interpreted as evidence of population-level reduction. Importantly, no dengue cases were reported during the study period. In a field trial in Petatlan, Mexico (2013–2014), a 71% reduction in *Aedes* oviposition was observed at the experimental site, using modified ovitraps (unpublished report, Ulibarri et al.) These findings align with the results of our study, where over 119,000 immatures were destroyed using just 40 ovillantas over 53-weeks. The larger volume and dark interior of the ovillanta provide a more appealing oviposition site, resulting in higher egg counts and improved sensitivity for vector surveillance programmes.

The ovillanta offers some practical and operational advantages over conventional ovitraps for mosquito surveillance and control. Because it is constructed from used tyres, the ovillanta is more durable, cost-effective, and environmentally sustainable than standard plastic ovitraps, which often degrade quickly under outdoor conditions. The design also reduces exposure to direct sunlight, and prevents rapid evaporation, all common limitations of conventional small plastic ovitraps. The curved inner surface of the tyre provides a naturally shaded, humid micro-environment that closely mimics the natural oviposition habitat of *Aedes* mosquitoes, thereby enhancing egg-laying attraction and overall trap productivity. Another significant advantage is the presence of an integrated drainage mechanism, which enables the easy collection of larvae and the reuse of the attractant solution. This reuse of infusion reduces labour, conserves resources, and maintains a stable semio-chemical environment that becomes increasingly attractive over time due to the accumulation of microbial volatiles. Repeated use of the same infusion likely increased its attractiveness to gravid *Aedes* females due to the buildup of olfactory cues contributing to a high number of larvae collected compared to conventional ovitraps that typically use fresh infusion each cycle.

Compared to the previous ovillanta trial at Guatemala, which primarily focused on collecting and destroying of *Aedes* eggs (Ulibarri et al., 2017), the present study introduces a novel operational approach targeting the destruction of immature mosquito stages. The trial utilized 40 ovillantas managed through mechanical removal of immature forms, ensuring disruption of the mosquito life cycle before adult emergence. This method enhances the eco-sustainability of vector control by reusing waste materials but also provides a direct and measurable impact on vector population dynamics. The observed significant reduction in immature densities compared to control sites indicates that the modified ovillanta strategy may have applicability as a practical and scalable tool for *Aedes* population suppression and dengue prevention.

We acknowledge that the study did not include direct comparison with other mosquito control tools or oviposition traps with mechanisms, similar to the ovillanta, which limits our ability to assess its relative effectiveness. Although the same study sites were monitored during both the pre- and post-intervention phases, variations in environmental conditions such as rainfall, temperature, and humidity, as well as human activities including water storage behaviour, waste management, and other vector control efforts, may have influenced the immature and adult mosquito densities independently of the intervention. Despite these limitations, the study provides preliminary evidence on the potential use of ovillantas as a supplementary tool for *Aedes* mosquito control, warranting further controlled and comparative investigations.

In this study using ovillanta ovitraps, only *Aedes* immatures were recorded, with no *Culex* species observed. The ovillanta trap is specifically designed to attract container-breeding *Aedes* mosquitoes by mimicking their preferred breeding sites. In contrast, *Culex* mosquitoes breed in a wide variety of habitats and typically favour larger, and more organically rich, or polluted water bodies. Therefore, they are less likely to be attracted to ovillanta traps. The absence of *Culex* in our collections likely reflects a reduced attraction to the trap conditions rather than their true absence in the study area. Another possible reason for lack of *Culex* in our study may be the selective attraction of the hay infusion–based ovillanta trap rather than their actual absence in the study area. Hay infusions are well known to be highly effective in attracting container-breeding *Aedes* mosquitoes by providing specific olfactory cues that stimulate their oviposition behaviour. In contrast, these cues may be less attractive to *Culex* species, which typically prefer more organically enriched or polluted water sources. However, the potential of the ovillanta to attract additional mosquito species, such as *Culex*, can be further explored in Zika- or West Nile–endemic areas by modifying the attractant solution used in the trap. Previous studies have shown that plant-based infusions effectively attract *Culex* mosquitoes (Barbosa et al., 2010; Wondwosen et al., 2024), suggesting that, with appropriate lure adjustments, the ovillanta could serve as a versatile tool for the surveillance and control of *Aedes* as well as *Culex* mosquito vectors.

This field trial provides evidence on the use of ovillantas in reducing vector populations. Ovillantas, are economical, environmentally friendly, and highly suitable for urban vector control. They may serve as a valuable supplementary tool in integrated vector management (IVM) programmes targeting *Aedes*-borne diseases. Successful implementation of this strategy would depend on sustained community engagement and participation.

## CRediT authorship contribution statement

**Abidha Suresh:** Writing – review & editing, Methodology, Investigation, Formal analysis, Data curation, Conceptualization. **Shriram Ananganallur Nagarajan:** Writing – review & editing, Supervision, Investigation, Conceptualization. **Jessu Mathew:** Writing – review & editing, Methodology, Investigation. **Amju Kolakattum Poyilthazham:** Methodology, Investigation. **Sonia Thankachy:** Writing – review & editing, Methodology, Investigation. **Prasanta Saini:** Investigation. **Kannan Thiruvengadam:** Formal analysis. **Pradeep Kumar Narendran:** Writing – review & editing, Conceptualization. **Ashwani Kumar:** Writing – review & editing, Investigation.

## Funding

The authors reported that this study was supported by internal funding from ICMR-NIVCR (Formerly ICMR-VCRC) for conducting the research presented in this article.

## Declaration of competing interest

The authors declare no conflict of interest. All co-authors have seen and agree with the contents of the manuscript and there is no financial interest to report. We certify that the submission is original work and is not under review at any other publication.

## Data Availability

Data will be made available on request.
